# Which Positions Optimize Pelvic Floor Activation in Female Athletes?

**DOI:** 10.3390/life15010058

**Published:** 2025-01-06

**Authors:** Elena Sonsoles Rodríguez-López, Luz María Martín-Márquez, María Barbaño Acevedo-Gómez, África López-Illescas, María Benito-de-Pedro, Cristina Ojedo-Martín

**Affiliations:** 1 Physiotherapy and Health Research Group (FYSA), Department of Physiotherapy, Faculty of Health Sciences—HM Hospitals, University Camilo José Cela, Villanueva de la Cañada, 28692 Madrid, Spain; lmaria.martinm@alumno.ucjc.edu (L.M.M.-M.); acevedogomez.maria@gmail.com (M.B.A.-G.); africa.lopez@csd.gob.es (Á.L.-I.); mbenito@ucjc.edu (M.B.-d.-P.); cojedo@ucjc.edu (C.O.-M.); 2 HM Hospitals Health Research Institute, 28015 Madrid, Spain; 3 Sports Medicine Center, Department of Sport and Health, Spanish Sports Council, 28040 Madrid, Spain

**Keywords:** pelvic floor, electromyography, posture, sportswomen, rugby

## Abstract

Background/Objectives: Implementing and optimizing pelvic floor muscle (PFM) training programs is crucial for reducing the risk of dysfunctions, improving athletic performance, and enhancing quality of life for athletes. The aim of this study was to assess PFM activation in female athletes during postural challenges. Methods: An observational and descriptive study was conducted with twenty-five female rugby players. Surface electromyography was used to evaluate the PFMs in five different body positions under stable and unstable conditions. Results: The peak amplitude of the PFMs at baseline differed according to the physical demand of each position (*p* < 0.001). The lowest percentage activation was in the supine position (16.23%), while the highest recruitment was observed during the parallel squat (40.69%). The percentage of maximum voluntary contraction also differed according to each position (*p* = 0.001). The values were similar in supine position, side plank (104%), and standing position, being significantly lower (*p* < 0.05) than those reached during the off-knees quadruped hold (121.58%), full plank (121.97%), and parallel squat (151.40%); however, the values were comparable between stable and unstable positions (*p* = 1.000). Conclusions: Positions that challenge gravity and pelvic biomechanics, such as the squat, plank, and quadruped, facilitate greater activation of the PFMs. Contrary to previous recommendations, these exercises do not appear to have significant negative effects; therefore, consideration should be given to the implementation of these exercises for the management of pelvic floor dysfunction and as part of comprehensive exercise programs designed to improve overall core and pelvic floor function.

## 1. Introduction

The increasing recognition of the pelvic floor (PF) with respect to overall bodily well-being has led to a growing body of research exploring the relationship between PF awareness, strength, and the improvement of conditions such as urinary incontinence (UI), as well as the potential for its role in preventing sports-related injuries [1,2]. In recent years, there has been a notable increase in the participation of women in high-impact sports, with rugby being one of the most notable examples. This sport, characterized by its intensity and physical demands, requires considerable endurance, strength, and agility, making it an ideal setting for studying the effects of high-impact exercise on women’s health [3].

UI is defined as the involuntary loss of urine, commonly observed in women in the postpartum and postmenopausal periods, due to weakening of the PF muscles (PFMs). Recent studies have reported an increase in the prevalence of stress UI (SUI) among young nulliparous athletes, suggesting that factors related to intense physical activity may influence the functionality of the PF [4]. SUI symptoms are more frequent in young females who practice high-impact sports, with a prevalence that varies between 25.9% and 70% [5]. High-impact sports, such as rugby, involve sudden movements, jumping, and constant physical contact—factors that increase pressure on the PF. This sustained and repetitive pressure can predispose women to developing UI, a condition that significantly affects their quality of life. The sports context is associated with a higher incidence of this condition, likely due to intense physical exertion, increased intra-abdominal pressure (IAP), and substantial biomechanical stresses placed on the body [4].

Awareness of PF health and the implementation of specific training programs are essential to prevent and improve the symptoms of UI in women [6]. Strengthening the PF through specific exercises can improve muscle function and endurance, thus reducing the incidence of pelvic floor disorders (PFDs). Furthermore, educating athletes and the general public about the significance of preserving adequate PF health is crucial for promoting healthy and sustainable sports participation [7]. According to a systematic review [5], PFM training can greatly decrease the symptoms and occurrence of UI in women involved in high-level sports, while also enhancing PFM function in female athletes.

To design a specific and personalized training program according to the sport practiced by a woman, it is essential to identify the positions that demand greater basal tone or optimize maximum voluntary contraction (VMC) of the PF. This would enable women to appropriately augment the muscular strength of their PF to withstand the biomechanical stresses and physiological demands that they encounter routinely [8]. The aim of our investigation was to assess PFM activation in female athletes during postural challenges, with the intent of developing a structured and effective exercise regimen.

## 2. Materials and Methods

The research was conducted in a single session, during which surface electromyography (sEMG) measurements were obtained from the PFMs. The study protocol was reviewed and approved by the Ethics Committee of the University Camilo José Cela (code 18_FRIU, 22 January 2022; Madrid, Spain).

### 2.1. Participants

The study participants were nulliparous women aged 18 to 35 years old with at least 5 years of rugby experience, recruited from local teams through pre-workout information sessions. The inclusion criteria stipulated a homogenous training regimen to ensure sufficient, consistent, and prolonged exposure to high PF impact.

Individuals were ineligible to participate if they presented with allergies to heavy metals, a lower limb injury or length discrepancy exceeding 0.7 cm [9], a history of urogynecological or pelvic surgery, pelvic irradiation, hypermobility syndrome, or mobility impairments that could impact the morphology, stiffness, or contractility of the PFMs. Additionally, pregnant women, and those with urgent UI, pelvic organ prolapse (POP), active or recurrent genitourinary tract infections, or a phobia of or inability to insert an intravaginal probe due to vaginal hypertonia [10], were also excluded from the study.

The female rugby players were provided with comprehensive details regarding the study, including the potential risks and benefits, and they granted both verbal and written informed consent before participating. Furthermore, all the data were managed anonymously and confidentially, in adherence to European data protection guidelines.

### 2.2. Instrumentation and Data Collection

The participants completed a questionnaire, which collected their anthropometric data, medical history, contraceptive use, smoking status, and details about their sports practice routine.

The activity of the PFMs was measured using an sEMG mDurance^®^ (Granada, Spain), a scientifically validated tool in the field of physiotherapy and sports rehabilitation that combines three parts: a portable sEMG, an operating system, and cloud analysis [11,12]. For all sEMG data, the sampling frequency was 2 kHz with an amplification gain of 1000, and the signals from the two devices were wirelessly sent to a computer (via Bluetooth) using two sensors, in order to record and evaluate the sEMG and range of movement [13,14].

All the participants received instructions on correct activation from an experienced physiotherapist before the sEMG assessment [15,16]. When participants did not correctly perform contraction, they were taught through physical examination and ultrasound imaging [17]. Then, verbal instruction for the voluntary contraction of the PFMs was given to them [13]. To achieve adequate contraction of the PFMs, audio logs were recorded to guide each participant’s tone, rhythm, and duration in the test, in order to provoke the same stimulus [18].

A Periform^®^ single-use vaginal probe (Neen HealthCare, Dereham, UK) with two electrodes, two independent tracks, one channel, and a total weight of 16 g, was employed for this purpose. The probe’s curved design allows it to be securely positioned against the lateral vaginal walls, facilitating the recording of activity in the nearby PFM. Anti-allergic gel was used by a specialist physiotherapist to introduce the vaginal probe, with the probe’s metal sensors at 3–9 o’clock. A reference electrode was placed on the anterior superior iliac spine, in accordance with SENIAM recommendations [19].

The adapted Glazer Protocol [20] was used to collect the electrical activity of the PFMs. First, the patient rested for 10 min in supine position, after which the following measurements were taken [13,21]: (i) Baseline in supine—once during 60 s of rest, we measured the average muscle activity at rest; (ii) VMC in supine—two VMCs of the maximum strength of the PFMs were measured: each was held for 5 s, followed by 30 s of relaxation [22]. The peak amplitude was the highest value of the two VMCs, in microvolts (μV). The reference value set as 100% for sEMG normalization was the VMC in supine position.

**Figure 1:**
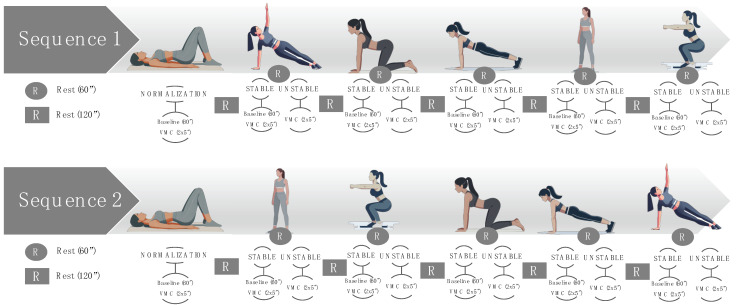
Sequences of multi-position measurements under stable and unstable conditions. “x” represents number of contractions, for example, 2x5” are 2 contractions of 5 seconds.

Measurements of basal and VMC activity were also taken later in five body positions under stable and unstable conditions. The values are represented in μV and percentages, according to the data normalized with respect to supine position. The initial measurement entailed a 60 s recording of the basal activation of the PFMs in each position, followed by VMC with a 60 s rest period between stable and unstable conditions and 120 s between positions. The measurements were conducted in a standardized manner, and the remaining four positions were randomized using the randomizer.org software (version 4.0), in order to obtain two different random sequences (Figure 1). The randomizer.org tool was also used to find out which sequence corresponded to each participant: (i) Sequence 1—side-lying position, quadruped, plank, standing position, and squat; (ii) Sequence 2—standing position, squat, quadruped, plank, and side-lying position.

Each position was performed in stable and unstable conditions: (i) stable side-lying position, with lateral support of the body on the floor; (ii) unstable side-lying position, side plank; (iii) stable quadruped, with support of the knees on the floor; (iv) unstable quadruped, off-knees quadruped hold; (v) stable plank, modified plank on knee; (vi) unstable plank, full plank; (vii) stable standing, with support of the back on the wall; (viii) unstable *standing*, without support; (ix) stable squat, or wall squat with back supported against the wall; and (x) unstable squat, parallel squat.

### 2.3. Statistical Analysis

The Gpower software 3.1 (Kiel University, Kiel, Germany) was used to calculate the sample size. Then, repeated measures (10 measurements), with an effect size of 0.80, an α error probability of 0.05, and statistical power of 0.90, were employed for the sample size calculation. The study required a sample size of 26 participants, based on the specified parameters. Sample sizes used in previous similar research were also taken into account [7].

The data were analyzed using the IBM Statistics Package for Social Science v.26 (IBM Corp, New York, NY, USA). The data are reported as the mean and standard deviation. The normality of the variables was assessed using the Shapiro–Wilk test. Multi-position sEMG data were analyzed with the Friedman test. To compare differences between positions, the Games–Howell post hoc test was performed. A confidence level of 95% was applied, with significance set at *p* < 0.05.

## 3. Results

The study was carried out on 29 female rugby players, 4 of whom were excluded from the analysis due to vaginismus and the impossibility of inserting the intracavitary probe. The final sample consisted of 25 nulliparous and eumenorrheic women with a mean age of 25.2 (3.52) years and a BMI of 24.2 (2.6) kg/m^2^.

**Figure 2:**
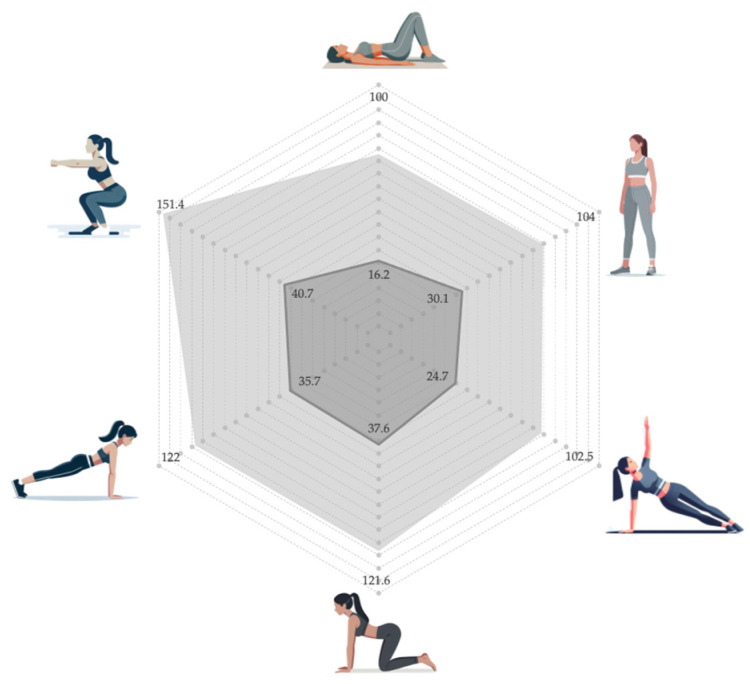
Comparison of the percentage of basal (dark gray) and VMC (light gray) activation levels in different unstable positions.

The peak amplitude of the PF at baseline differed according to the physical demand of each position (*p* < 0.001), as represented in Table 1. The lowest percentage activation was in the supine position, while the highest recruitment was during the parallel squat (unstable). The baseline percentage was below 20% in supine position; around 20–30% in standing position and side plank; and above 30% in quadruped hold, plank, and squat (Figure 2). Moreover, there were statistically significant differences between these three groups, according to percentage activation (*p* < 0.001). Although activation increased slightly with instability in all positions except the plank, the baseline of the PF was similar (*p* = 1.000), according to the instability challenge in all the positions. The baseline values for each position were similar in the two randomized sequences (*p* > 0.05).

The peak amplitude of VMC also differed according to the physical demand of each position (*p* = 0.001), as represented in Table 2. The lowest VMC was observed for the side plank, while the highest recruitment occurred during the squat. The activation values of VMC were similar in the supine, side plank, and standing positions (*p* = 1.000), being significantly lower (*p* < 0.05) than those reached during the off-knees quadruped hold (121.58%), full plank (121.97%), and parallel squat (151.40%) (Figure 2). The VMC for each position was similar in the two randomized sequences (*p* > 0.05).

## 4. Discussion

This observational study aimed to evaluate PFM activation in female athletes during postural challenges. The International Consultation on Incontinence classifies the effectiveness of Kegel’s PFM exercises as level “A” evidence in evidence-based medicine [6]. Therefore, it is crucial to determine which postures are most effective for activating the PF, in order to develop a structured and efficient exercise regimen based on PF activation. The findings suggest that the baseline activity of the PFMs is comparable under both stable and unstable conditions. However, baseline and voluntary activation were found to differ according to the physical demand of each position. The parallel squat, followed by the full plank and the off-knees quadruped hold, seem to induce the highest activation of the PFMs in rugby players for both baseline and VMC.

Numerous studies have documented variations in the basal activity and/or activation of the PFMs when comparing supine and upright positions. Gimenez M. et al. [23] concluded that basal activity was 36% higher when standing than when lying down; our data also reflected an increase in basal activity when standing, although only by 10%. They also showed that, when standing, the maximum contraction was lower than in supine position; however, in our study, a slight increase in VMC was observed when assuming a bipedal posture. Other studies using manometry have also seemed to report increased PFM strength in the standing position [8,24,25]. In contrast, Madill and MacLean found no differences between supine, sitting, and standing positions [26]. However, it is difficult to compare our results with sEMG, as manometry uses a vaginal pressure transducer that may be impacted by IAP increases, such as those observed when standing. This finding may be related to the activation of type II (fast) fibers, which are quickly activated voluntarily due to an increase in IAP [27], likely related in collateral manner to the physical demand associated with rugby. Another potential hypothesis regarding the effect of exercise on the PF is the role of gravity and the resulting increase in IAP. Our study emphasizes the influence of positions that leverage gravity, assisting women in activating the PFMs and causing a stronger involuntary contraction. This correlates with other research which states that exercise position during a test is important, as standing is associated with increased IAP, leading to involuntary contraction of the PFMs [28,29]. This could be linked to greater stimulation of the PFMs, leading to enhanced proprioception. This statement may contradict recommendations to perform exercises without increasing IAP, suggesting that such exercises do not have a negative impact. Conversely, it should be noted that traditionally extended positions for training this musculature—such as the supine and side-lying positions—show minimal relevance in terms of activation, with lower activation data. Authors including Rodríguez-Mias et al. [30] found no differences in prolapse measurements between lying and standing positions. Additionally, there appears to be growing interest in developing protocols for the standing position, in which the true mechanism of PF function can be better understood [31].

In terms of studies examining myoelectric activity changes in the PFMs based on posture, Sapsford et al. [32] found that unsupported sitting postures—whether upright or high—demanded more PFM activity compared to supported sitting postures. A systematic review [33] revealed that the PFMs exhibit significantly higher resting activity in a neutral ankle position and at 15° dorsiflexion, compared to 15° plantar flexion. These results correlate with those of Lee et al., affirming that a more unstable posture induces greater co-activation of the trunk stability muscles and PFMs [34].

Dayican et al. [35] have assessed the PFMs using sEMG in three positions: modified butterfly pose, modified child pose, and modified deep squat with block pose. They showed that healthy PFMs contracted maximally in modified butterfly pose, the position in which they were most relaxed; however, these findings differed in the case of females with PFD. In our study, we did not distinguish between women with PFD, and so we could not confirm whether activation levels varied based on the functional state of the PFMs. However, our findings are consistent with the increased activation reported in unsupported or unstable postures.

Díaz-Mohedo et al. [7] analyzed PFM activity during various functional movements, finding that the trunk stability push-up elicited the highest PFM activation, compared to other movements. In contrast, our study showed greater activation during the parallel squat, which is similar to their overhead squat. Additionally, the movements in their study involved a more significant increase in IAP than the stable and unstable postures used in our study, potentially contributing to the variability observed in the results. In another study [36], researchers analyzed PFM-related myoelectric activity during Modified Pilates exercises, concluding that the greatest activation occurred during core (treaser) and plank positions. Their results can be considered similar to ours, as they did not describe any exercise such as the parallel squat.

Therefore, to induce greater activation of the basal tone, work in positions such as squat, plank, and quadruped should be alternated, especially for those playing sports that require explosiveness and impact, such as rugby, in which high-intensity, short-duration challenges are posed (e.g., jumps, tackles, plyometrics, decelerations). In turn, for exercises that aim to address challenges of low intensity and long duration, planks or quadruped exercises should be implemented, especially those that start from unstable conditions.

For female athletes, a strategy that progresses from easier to more challenging exercises, with increasing activation, could be effective in promoting better adherence and treatment outcomes [37,38]. This approach would begin with supine or side-lying position, in order to help develop awareness of the PFMs where demand and muscle fiber recruitment are lower [39]. Subsequently, the progression would involve transitioning to a standing position to intensify the challenge through gravitational forces, followed by the inclusion of controlled stable exercises, such as planks. Ultimately, quadrupedal movements and squats would be integrated into the regimen. As a possible suggestion, it would be convenient to broaden the spectrum of PF activity applied to movements that are adapted to the sporting gesture, following a progression of exercises such as those reported in this study. Implementing individualized programs can improve the quality of training in female athletes [40]. Strengthening the PFMs can greatly decrease the likelihood of incontinence episodes, enabling athletes to enjoy a more comfortable and secure lifestyle. This improvement enhances their overall well-being and boosts their confidence in both daily and sporting activities [41].

As for limitations, although the positions of each sequence and the assignment to each player were randomized, it should be noted that all the measurements were made on a single day. For this reason, it is possible that accumulated muscle fatigue may have influenced the final positions, even though the values for each position were similar in the two random sequences. While the sample was homogeneous, focusing on a single sport, the small sample size necessitates cautious interpretation of the findings. A larger sample would yield more representative data regarding the characteristics of the study population and the specific sport examined. As the measurements were conducted in static positions, it would be advisable to incorporate protocols evaluating more dynamic or habitual positions associated with each sport’s practice [40]. As a future line of research, we consider that further long-term research based on randomized clinical trials is necessary to assess the effect that the progression of postures can have on pelvic floor strength.

## 5. Conclusions

According to traditional recommendations for PFM training programs, it is important to consider a gradual progression in the difficulty of the exercises, starting with less demanding positions (supine, standing, or side plank) and progressing to more complex challenges (quadruped hold, plank, and squat).

However, our findings indicate that the squat, followed by the plank and quadruped postures, yield the most robust PFM activation. These postures, by defying gravity and pelvic biomechanics, promote high muscle activation, which could improve proprioception and involuntary muscle response. Contrary to previous recommendations, these exercises do not appear to have significant negative effects on intra-abdominal pressure, suggesting that they may be beneficial for PF strengthening in athletes. For this reason, the addition of these exercises into programs for the management of pelvic floor dysfunction or comprehensive exercise programs designed to enhance overall core and PF function may be warranted. Correct technique and supervision by a trained professional are essential components of an effective and individualized pelvic floor exercise program.

## Figures and Tables

**Table 1:** Peak amplitude of baseline data in different positions and conditions.

Position	Condition	Exercise	Peak Amplitude (in μV)					Peak Amplitude (in %)				
			Mean	SD	95% CI		*p* Value *	Mean	SD	95% CI		*p*-Value *
Supine (Normalization)	Stable	Supine	23.42	9.31	15.34	25.09	<0.001	16.23	12.11	11.67	24.27	<0.001
Standing	Stable	Standing with support	39.27	13.35	33.75	44.78		28.46	20.11	20.16	36.76	
	Unstable	Standing	42.48	19.52	34.42	50.54		30.15	19.67	22.03	38.27	
Side-lying	Stable	Side-lying position	26.84	16.42	20.06	33.62		22.03	24.44	11.94	32.12	
	Unstable	Side plank	31.69	17.92	24.29	39.09		24.69	22.42	15.44	33.95	
Quadruped	Stable	Quadruped hold	40.39	14.83	34.27	46.51		29.64	21.18	20.89	38.38	
	Unstable	Off-knees quadruped hold	56.72	60.75	31.65	81.80		37.59	30.73	24.91	50.27	
Plank	Stable	Plank on knee	57.06	26.73	46.03	68.09		39.49	24.41	29.41	49.56	
	Unstable	Full plank	52.11	23.29	42.50	61.72		35.71	20.26	27.35	44.07	
Squat	Stable	Wall squat	47.52	25.89	36.84	58.21		38.13	41.86	20.85	55.41	
	Unstable	Parallel plank	53.53	19.69	45.40	61.66		40.69	33.68	26.78	54.59	

CI, confidence interval; SD, standard deviation. * Significance level was set at *p* < 0.05, based on Friedman test.

**Table 2:** Peak amplitude of VMC data in different unstable positions.

	Peak Amplitude (in μV)					Peak Amplitude (in %)				
	Mean	SD	95% CI		*p* Value *	Mean	SD	95% CI		*p*-Value *
Supine (Normalization)	169.08	72.12	139.31	198.85	0.001					0.001
Standing	169.13	80.34	135.97	202.29		104.00	32.40	90.63	117.38	
Side plank	163.93	74.55	133.15	194.70		102.49	30.15	90.04	114.93	
Quadruped hold	192.96	79.99	159.95	225.98		121.58	30.81	108.86	134.29	
Full plank	193.56	82.35	159.57	227.56		121.97	41.79	104.73	139.22	
Parallel squat	208.10	70.30	179.08	237.12		151.40	109.4	106.24	196.56	

CI, confidence interval; SD, standard deviation. * Significance level was set at *p* < 0.05, based on Friedman test.

## Data Availability

The data presented in this study are available upon request from the corresponding author. The data are not publicly available due to privacy and ethical restrictions.
